# Effect of Defatting Method on the Nutritional, Functional, and Bioactive Properties of Black Soldier Fly *(Hermetia illucens)* Larvae

**DOI:** 10.3390/insects16080844

**Published:** 2025-08-15

**Authors:** Natasha Spindola Marasca, Alan Carvalho de Sousa Araújo, Karoline da Silva Noda, Bruna Silva de Farias, Ana Paula Dutra Resem Brizio, Sibele Santos Fernandes, Vilásia Guimarães Martins

**Affiliations:** 1 Laboratory of Food Technology, School of Chemistry and Food Engineering, Federal University of Rio Grande (FURG), Av. Itália, Km 8, Carreiros, Rio Grande 96203-900, RS, Brazil; natasha.spindola@hotmail.com (N.S.M.); karol.noda1910@gmail.com (K.d.S.N.); bruna.farias.furg@gmail.com (B.S.d.F.); anabrizio@yahoo.com.br (A.P.D.R.B.); vilasiamartins@gmail.com (V.G.M.); 2 Laboratory of Functional Biochemistry of Aquatic Organisms, Institute of Oceanography, Federal University of Rio Grande (FURG), Av. Itália, Km 8, Carreiros, Rio Grande 96203-900, RS, Brazil; alandesousa02@hotmail.com

**Keywords:** antioxidant activity, defatting methods, edible insects, phytochemical screening, protein enrichment

## Abstract

Defatting methods are key to modulating the nutritional, functional, and bioactive characteristics of edible insect powders. This study evaluated the effects of mechanical pressing and ethanol-based solvent extraction on *Hermetia illucens* larvae powder. Solvent-defatted samples (DPSs) showed the highest protein content (54.96 g/100 g), with a 61% increase compared to full-fat powder (FP), and the lowest residual lipid content (3.18 g/100 g). In contrast, mechanical pressing (DPP) preserved higher antioxidant activity (68.30% DPPH inhibition), a 30% increase over FP. DPS also showed greater fiber content (13.90 g/100 g), improved water solubility, emulsification capacity, and reduced water activity (0.269), desirable traits for food formulations. DPP retained higher hygroscopicity and exhibited the highest antioxidant potential among the samples. These findings demonstrate that defatting method selection significantly impacts the techno-functional and nutritional quality of insect powders and should align with the desired end use, whether for protein enrichment, enhanced antioxidant activity, or development of sustainable food ingredients. This work supports the strategic use of *Hermetia illucens* as a functional, high-protein ingredient and reinforces its role in advancing circular and sustainable food systems.

## Simple Summary

Edible insects are gaining attention as a sustainable alternative to traditional animal protein sources. Among them, the black soldier fly (*Hermetia illucens*) stands out due to its high nutritional value and low environmental impact. However, to improve the quality of insect-based powders used in food products, it is important to remove part of the fat. In this study, we compared two ways to reduce fat from the larvae: mechanical pressing and a method using alcohol. We found that removing fat significantly changes the powder’s properties. The alcohol-based method increased protein levels and improved how well the powder dissolves and mixes with water, features that are useful in making food products. On the other hand, mechanical pressing kept more natural antioxidant compounds, which can offer health benefits and help preserve food. The choice of fat removal method can make the powder better suited for different food applications, whether the goal is to add protein or to enhance health-promoting properties. This research helps improve the use of insects in food and supports the development of more sustainable and nutritious products.

## 1. Introduction

The United Nations projects that the world population will grow from seven to nine billion by 2030, with 60% of the population expected to migrate to urban areas [1]. This rapid population growth, combined with increased urbanization, raises serious concerns about global food security [2]. One of the most pressing challenges will be ensuring an adequate supply of protein, an essential nutrient for human health, which has led to the search for alternatives to traditional sources. In response, edible insects, particularly black soldier fly larvae, *Hermetia illucens* (BSF), have been proposed as promising food alternatives to address economic, environmental, and health challenges [3]. The industrial production of BSF has expanded with companies such as AgriProtein, Ynsect, Enviroflight, and HaoCheng Mealworms leading the way [4].

The European Food Safety Authority (EFSA) has evaluated BSF as a novel food ingredient, highlighting its potential for food sector innovation [5]. BSF larvae efficiently convert organic materials, such as agro-food leftovers, animal manure, and urban waste, into biomass rich in proteins and lipids [6,7,8]. Nutritionally, BSF offers high protein (30–53 g/100 g dry base), lipids (20–41 g/100 g), calcium (840–934 mg/100 g), iron (2.1–3 mg/100 g), and zinc (6.8–15 mg/100 g) [9,10].

Food neophobia remains a significant challenge for the acceptance of edible insects, particularly in Western and urban populations [11]. However, Schösler et al. [12] suggested that this resistance can be mitigated when insects are incorporated into foods in the form of processed ingredients such as flour, powder, or paste. The use of these ingredients in widely consumed foods, such as bakery products, snacks, and meat analogues, can facilitate the acceptance of insects as part of the diet, as their incorporation helps to overcome sensory and cultural barriers that lead to the rejection of insects in their natural form. Several studies have demonstrated the successful incorporation of insect powders into food formulations, including breads, pasta, burgers, and protein bars [13,14,15]. Moreover, consumer studies have shown that familiarity, product format, and information about sustainability and health benefits can positively influence acceptance [16,17]. Insect powder has emerged as a promising approach for the integration of edible insects into food systems.

Edible insects are marketed in various forms, including whole insects, pastes, ground flours, protein powders, and oil fractions, and are widely used as ingredients in the food industry [18,19,20]. Various methods can be employed for defatting insects; however, the quality of the final product is directly influenced by the type of procedure used, whether for obtaining oil or protein [21]. Non-defatted insects, especially in flour form, tend to present lower oxidative stability due to their high lipid content, which can lead to rancidity and off-flavors during processing and storage [22,23]. These limitations can compromise shelf life and sensory quality, restricting their application in certain food products [22,24]. Given this scenario, defatting has been proposed as a strategy to improve the oxidative stability and functional performance of insect-based ingredients [19,23]. Therefore, large-scale industrial development requires a comprehensive analysis of the nutritional, techno-functional, and antioxidant properties of edible insects as food ingredients to ensure their viability and functionality in products intended for human consumption [5,25].

High lipid content in insect powders can negatively affect their physicochemical and functional properties. Excess fat may promote oxidative instability, reduce shelf life, interfere with protein solubility, and impair emulsifying and hydrating capacities, limiting their potential in food applications. Defatting has therefore become a key strategy to improve the nutritional profile, enhance functional attributes, and ensure product stability. By removing lipids, the protein concentration increases and the matrix becomes more hydrophilic, which favors water interaction and ingredient performance in formulations such as meat analogues, baked goods, and beverages [21,26]. Defatting can be achieved by using organic solvents or mechanical processes. However, concerns related to food safety, environmental impacts, and health risks have intensified the debate on the feasibility of these processes at an industrial scale [26]. In addition to solvent-based and mechanical defatting, other methods have been explored in the literature, including aqueous extraction and supercritical CO_2_. These techniques have shown promising results in specific applications due to their selectivity, efficiency, or environmental performance. However, their use remains limited by high operational costs, scalability issues, or regulatory restrictions in food-grade processing [21,26].

Among organic solvents, ethanol is a safe alternative and has been widely studied for lipid removal from various food matrices, including soybeans [27], ground corn [28], and other sources intended for human consumption [29]. Additionally, ethanol has shown high efficiency as an extraction agent for defatting raw food materials, and is particularly relevant for subsequent protein extraction processes [21].

Mechanical pressing is a widely used technique in several countries due to its efficiency, low initial costs, and operational simplicity. This method has been widely adopted by farmers. Despite the emergence of more innovative extraction technologies, mechanical pressing preserves the nutritional properties of the extracted oils [30]. Furthermore, it does not require the use of organic solvents to separate the oil, making it particularly effective in extracting oils from oilseeds, such as soybeans, cotton, and olives. Although its extraction efficiency is considered relatively low [31], mechanical pressing has been applied to obtain oils from insects with a high lipid content (20% c/w or higher), such as *Tenebrio molitor* and *Hermetia illucens* [32,33].

Given the increasing interest in edible insects as sustainable protein sources, this study investigates how mechanical pressing and solvent-based defatting modulate the nutritional, functional, and bioactive characteristics of *Hermetia illucens* powder. The findings offer insights into processing strategies that improve protein concentration and preserve bioactive compounds, supporting the integration of insect-based ingredients into innovative and sustainable food systems.

## 2. Materials and Methods

### 2.1. Materials

BSF larvae in full powder form (FP) were acquired from a specialized company (Kimmy Organic Farm, Ho Chi Minh City, Vietnam). Both the mechanically defatted powder (DPP) and the solvent-defatted powder (DPS) were obtained from this same FP sample, which originated from a single batch of insects, and DPPH (2,2-diphenyl-1-picrylhydrazyl) and ABTS (2, 2′-azinobis (3-ethylbenzothiazoline-6-sulphonic acid) were acquired from Sigma Aldrich (St. Louis, MO, USA). All reagents used in the chemical analyses were of analytical grade.

### 2.2. Methods

#### 2.2.1. Defatting

The defatting of the full powder form (FP) using ethanol was performed according to the methodology of Zhao et al. [21] with modifications. Ethanol (99.5%) was used as the extraction solvent at a ratio of 5 mL/g of whole powder at 40 °C, with a stirring time of 60 min in a shaker (Cientec, model CT-712RNT, São Paulo, Brazil). The mixture was then filtered using a filter cloth, and the process was repeated for two cycles. After two extraction cycles under the aforementioned conditions, the filtered material was dried in an oven (Fanem, model 515-C, São Paulo, Brazil) under forced air ventilation at 60 °C for 4 h. Subsequently, the defatted powder (DPS) was packed in polyethylene bags and stored at −18 °C until further use. The defatting of the full powder form (FP) by mechanical pressing was carried out using a cold-pressing method, resulting in the defatted powder by pressing (DPP). This process was performed at the industrial level by the supplier (Kimmy Organic Farm), and the powder was packed in polyethylene bags and stored at −18 °C until further use.

#### 2.2.2. Physicochemical and Thermal Characterization of FP, DPP, and DPS

##### Proximal Composition and Caloric Value

The FP, DPP, and DPS samples were subjected to proximate compositional analyses. Moisture (No. 960.39), ash (No. 923.03), and protein content using the Kjeldahl method (No. 992.15) and lipids were determined using the Soxhlet method (No. 925.30) according to AOAC [34]. A conversion factor of 5.6 was used according to Janssen et al. [35] to convert nitrogen into protein. The fiber content was determined using the methodology of Instituto Adolfo Lutz [36]. The caloric values of FP, DPP, and DPS were calculated using the average values of the protein, carbohydrate, and lipid coefficients [37], as shown in Equation (1).

Caloric value (kcal/100 g) = (g de protein × 4) + (g de lipids × 9) + (g de carbohydrates × 4)(1)

##### Water Activity (Aw)

The water activity of BSF samples was determined using a LabTouch Novasina^®^ water activity analyzer (Novasina, Model LabTouch, Lachen, Switzerland) at room temperature (~25 °C).

##### Color Properties

The color parameters of FP, DPP, and DPS were measured using a Colorimeter (Minolta, model CR-400, Tokyo, Japan). The analyses were based on the CIEL*a*b* system, where L* = brightness, a* = −green/+red, and b = −blue/+yellow. The hue angle (h°) was calculated using Equation (2).

(2) $$h{}^{\circ}=\tan^{-1}\frac{b^{*}}{a^{*}}$$

##### Differential Scanning Calorimetry (DSC)

The thermal characteristics of BSF samples were determined according to the methodology of Lucas et al. [38] by DSC (Shimadzu, model DSC-60, Kyoto, Japan). DSC analysis was performed at a heating rate of 10 °C/min in the temperature range from −25 to 300 °C, and a nitrogen atmosphere flow rate of 50 mL/min. Approximately 2 to 3 mg of sample was weighed into a sealed aluminum pan, and an empty aluminum pan was used as the reference. The analyzed thermal parameters included onset temperature (T_0_), peak temperature (T_p_), final temperature (T_f_), and enthalpy change (ΔH) of thermal transitions.

#### 2.2.3. Functional and Technological Properties

##### Water- and Oil-Holding Capacity

The water-holding capacity (WHC) and oil-holding capacity (OHC) were analyzed following the methodology of Santos and Martins [39]. Briefly, 0.5 g of flour was mixed with 20 mL of distilled water or soybean oil (1:40, *w*/*v*) in tared centrifuge tubes (Wi), vortexed for 2 min, left to rest for 30 min, and centrifuged (Biosystem, model LPW350, Campinas, Brazil) at 8709× *g* for 10 min. The supernatant was discarded and the tubes with the absorbed samples were weighed (Wf). The WHC and OHC were calculated using Equation (3).

(3) $$\frac{WHC}{OHC}\;\left(\%\right)=\left(\frac{Weight\;of\;the\;hydrated\;sample\;\left(g\right)-Weight\;of\;the\;dry\;sample\;\left(g\right)}{Weight\;of\;the\;dry\;sample\;\left(g\right)}\right)\times 100\;\;\;\;\;\;\;\;\;$$

##### Emulsification Capacity and Stability

The emulsifying capacity (EC) and emulsion stability (ES) were determined following the methodology described by Bento et al. [40]. The sample (0.35 g) was weighed into a 10 mL centrifuge tube, followed by the addition of distilled water (2.5 mL). The tubes were vortexed for 30 s, and corn oil (2.5 mL) was added. Next, the tubes were vortexed again for 90 s and centrifuged (Biosystem, model LPW350, Brazil) at 500× *g* for 5 min. The emulsifying activity was determined by the ratio of the volume of the emulsified layer to the total volume before centrifugation. The emulsion stability was evaluated using the same procedure; however, before centrifugation, the samples were subjected to heat treatment at 85 °C in a thermostatic bath for 15 min and centrifuged (Biosystem, model LPW350, Brazil) at 500× *g* for 5 min. The EC and ES were calculated using Equations (4) and (5), respectively.

(4) $$\mathrm{EC}\;(\%)=(\frac{Volume\;of\;the\;emulsified\;layer\;(mL)}{Total\;volume\;before\;centrifugation\;(mL)})\times 100$$

(5) ES (%)= % of the remaining emulsifying activity after heating

##### Water Absorption Index and Swelling Capacity

Gel hydration parameters, including the water absorption index (WSI) and swelling capacity (SC), were determined according to Torbica et al. [41] and Hong et al. [42], with minor adjustments. In a centrifuge tube, 0.25 g (dry basis) powder samples were dissolved in 5 mL deionized water and heated in a shaking water bath at 90 °C for 15 min and before being centrifuged (Biosystem, model LPW350, Brazil) for 10 min at 3000× *g* and 4 °C. The precipitate was weighed and the supernatant was placed in an aluminum capsule and evaporated at 105 °C until it reached a constant weight. The WSI and SC were calculated using Equations (6) and (7), respectively.

(6) $$\mathrm{WSI}=\frac{\mathrm{Weight}\;\mathrm{of}\;\mathrm{dissolved}\;\mathrm{solids}\;\mathrm{in}\;\mathrm{supernatant}\;(g)}{\mathrm{Sample}\;\mathrm{weight}\;(g)}$$

(7) $$\mathrm{SC}=\frac{\mathrm{Weight}\;\mathrm{of}\;\mathrm{the}\;\mathrm{sediment}\;(g)}{\mathrm{Sample}\;\mathrm{weight}-\mathrm{Weight}\;\mathrm{of}\;\mathrm{dissolved}\;\mathrm{solids}\;\mathrm{in}\;\mathrm{supernatant}\;(g)}\;$$

##### Hygroscopicity

Hygroscopicity was determined according to the method described by Fernandes et al. [43] with some modifications. Samples from each treatment (1 g) were kept in a vessel containing a saturated NaCl solution at 25 °C (75%, relative humidity—RH) for 1 week. The samples were then weighed, and the hygroscopicity (%) was determined as the mass (in g) of the water adsorbed per 100 g of dry solid. To verify the water absorption behavior of the samples, the amount of water absorbed was measured after 24 h and 7 days of exposure to a controlled environment. The hygroscopicity was calculated using Equation (8).

(8) $$Hygroscopicity\;\left(\%\right)=\left(\frac{Weight\;water\;absorbed\;\left(g\right)}{Weight\;dry\;solid\;\left(g\right)}\right)\times 100\;\;\;\;\;\;\;$$

#### 2.2.4. Bioactive Compounds

##### Antioxidant Activity

The antioxidant activity of the extract was analyzed following the methodology described by Vanqa et al. [5]. In total, 2 g of edible insect flour was mixed with 40 mL of Milli-Q water in a 50 mL centrifuge tube. The mixture was centrifuged at 25 °C for 15 min at 8709× *g* (Biosystem, model LPW350, Brazil), and the supernatant was collected and stored at 4 °C for further analysis. The antioxidant capacity was determined by the sequestration of the DPPH radical by Rufino et al. [44] and the ability to capture the ABTS radical by Herrero et al. [45]. The DPPH free radical scavenging capacity and capture of the ABTS radical were expressed according to Equation (9), where Abs control is the absorbance of the sample and Abssample is the absorbance without the sample.

(9) $$DPPH\;Radical\;Sequestration\;or\;ABTS\;radical\;capture\;\left(\%\right)\;\;\;=\;\frac{\left({Abs}_{control}-{Abs}_{sample}\right)}{{Abs}_{control}}\times 100$$

##### Phytochemical Screening

To recover the maximum amount of bioactive components, FP, DPP, and DPS samples (1:20, *w*/*v*) were extracted with distilled water, methanol (50%), and ethanol (50%), respectively, in a shaker (Cientec, model CT-712RNT, Brazil) at 150 rpm and 40 °C for 60 min. After extraction, the samples were centrifuged (Biosystem, model LPW350, Brazil) at 8709× *g* for 10 min. The supernatant was filtered using a filter paper and a vacuum pump. Extracts from BSF samples were stored at 4 °C for subsequent analysis [46].

Phytochemical screening of the different extracts obtained from the FP, DPP, and DPS samples was performed to confirm the presence of certain chemical families (coumarins, flavonoids, saponins, flavanones, steroids, tannins, quinones, and phenols). It was determined by solubility tests, color reactions with characteristic reagents, and precipitation. These tests were carried out on both aqueous and organic extracts according to Purewal et al. [47] and Purewal et al. [46].

In summary, coumarins were identified by the formation of a yellow color after reaction with sodium hydroxide (10%). Flavonoids were confirmed by the appearance of a yellow precipitate after the reaction with lead acetate. Saponins were detected by the formation of persistent foam after shaking with water. Flavanones were identified by the formation of a crimson red color after the slow addition of concentrated sulfuric acid. Steroids were confirmed by the formation of a red color in the chloroform-sulfuric acid layer. Tannins were revealed by the reaction with ferric chloride, resulting in a blue-black color.

#### 2.2.5. Statistical Analysis

All measurements in the present study were performed in triplicate. The results were analyzed by analysis of variance (ANOVA), and the statistical difference (*p* < 0.05) was analyzed by Tukey’s test using the software Statistica 5.0 (StatSoft, Tulsa, OK, USA).

## 3. Results

### 3.1. Physicochemical and Thermal Characterization of FP, DPP, and DPS

#### 3.1.1. Proximal Composition and Caloric Value

The proximal composition, caloric value, color, and thermal properties of *Hermetia illucens* powders subjected to different defatting methods are presented in Table 1. After the defatting process, a gross yield of 64% defatted flour was obtained. Moisture content varied among treatments, with the DPS sample showing the highest value (7.01 ± 0.32 g/100 g), followed by FP (6.07 ± 0.10 g/100 g) and DPP (5.20 ± 0.17 g/100 g). Protein content increased after defatting, reaching 54.92 g/100 g in DPS and 39.61 g/100 g in DPP, compared to the intact sample (FP).

Lipid content ranged from 32.45 ± 4.53 g/100 g (FP) to 3.18 ± 0.55 g/100 g (DPS), while DPP retained an intermediate value (21.70 ± 1.39 g/100 g). Ash content increased with defatting: 8.58 ± 0.08 g/100 g in FP, 9.42 ± 0.15 g/100 g in DPP, and 10.70 ± 0.04 g/100 g in DPS. Crude fiber was highest in DPS (13.90 ± 0.43 g/100 g), followed by FP (8.87 ± 0.62 g/100 g) and DPP (7.73 ± 0.35 g/100 g). Caloric values decreased with fat removal: FP presented the highest energy value (471.01 kcal/100 g), followed by DPP (407.37 kcal/100 g), and DPS the lowest (288.64 kcal/100 g).

#### 3.1.2. Water Activity (Aw)

Table 1 also shows the water activity (Aw) values of the samples. FP exhibited the highest Aw (0.571), followed by DPP (0.529) and DPS (0.269), indicating a reduction in water retention capacity with the use of solvent-based defatting.

#### 3.1.3. Color Properties

Colorimetric parameters (L*, a*, b*) are presented in Table 1. DPS showed a lighter color compared to FP and DPP. The a* value (redness) decreased from 9.54 (FP) to 5.92 (DPS), while b* (yellowness) increased progressively from FP (22.53) to DPP (23.40) and DPS (24.01). The hue angle (h°) also increased in DPS, indicating a shift toward a more yellowish tone.

#### 3.1.4. Differential Scanning Calorimetry (DSC)

Thermal analysis indicated that FP presented an endothermic peak above 100 °C, while DPP and DPS showed peaks below 100 °C. The DPS sample exhibited the highest enthalpy of water evaporation (ΔH = 108.66 J/g). The onset, initial, and final evaporation temperatures were lower in DPP, followed by DPS and FP.

### 3.2. Functional and Technological Properties

#### 3.2.1. Water- and Oil-Holding Capacity

**Figure 1:**
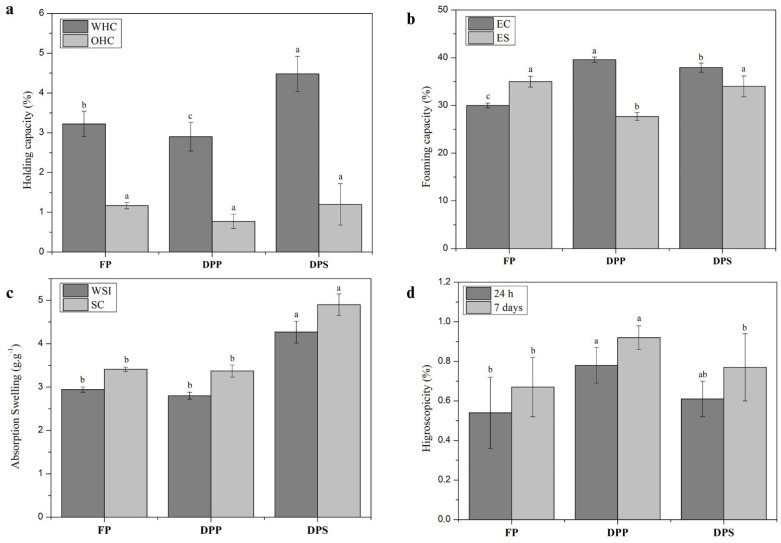
Technological properties of BSF samples. (**a**) Holding capacity; (**b**) emulsifying capacity; (**c)** absorption swelling; (**d**) hygroscopicity. FP = full powder form; DPP = defatted powder by pressing; DPS = defatted powder by solvent. WHC = water-holding capacity; OHC = oil-holding capacity; EC = emulsification capacity; ES = emulsification stability; WSI = water absorption index; SC = swelling capacity. Average of three values with standard deviation; different superscript letters are significantly different (*p* < 0.05).

The techno-functional properties of BSF powders are presented in Figure 1a. Water-holding capacity (WHC) differed among the samples. The DPS sample exhibited the highest WHC, while DPP showed the lowest. Oil-holding capacity (OHC) did not differ significantly among the samples.

#### 3.2.2. Emulsification Capacity and Stability

Emulsification capacity (EC) and emulsion stability (ES) are shown in Figure 1b. Both DPP and DPS demonstrated greater EC than the intact powder (FP), with DPS showing the highest capacity. Regarding ES, DPS (34.0%) and FP (35.0%) had slightly better stability than DPP.

#### 3.2.3. Water Solubility Index and Swelling Capacity

As shown in Figure 1c, the DPS sample exhibited higher values for both water solubility index (WSI) and swelling capacity (SC) compared to DPP and FP. These results indicate improved hydration properties following solvent-based defatting.

#### 3.2.4. Hygroscopicity

Figure 1d presents the hygroscopicity data. The DPP sample absorbed more moisture than DPS and FP after 24 h (0.78%) and after 7 days (0.92%). The lowest values were observed for FP. DPS showed intermediate hygroscopicity.

### 3.3. Bioactive Compounds

#### 3.3.1. Antioxidant Activity

Antioxidant activity of BSF powder samples was evaluated using ABTS and DPPH assays (Table 2). Both assays demonstrated that defatted samples (DPP and DPS) exhibited higher antioxidant capacity than the intact sample (FP). Among them, DPP showed the highest antioxidant activity.

#### 3.3.2. Phytochemical Screening

Preliminary phytochemical screening was conducted to identify secondary bioactive compounds in the BSF powders, and the results are presented in Table 3. The defatted samples (DPP and DPS) exhibited a greater presence of phytochemicals compared to the intact form (FP). Additionally, ethanol extracts showed higher yields of phytochemicals than methanol extracts. However, aqueous extracts also produced comparable or superior results in some cases.

Specific classes of compounds, such as flavonoids, coumarins, and flavanones, were detected primarily in the defatted samples. Tannins, quinones, and phenols were not detected in any sample, and steroids were identified only in aqueous extracts.

## 4. Discussion

### 4.1. Physicochemical and Thermal Characterization

The defatting method had a substantial impact on the chemical composition of *Hermetia illucens* powder. Solvent defatting (DPS) led to a more effective lipid removal, which in turn concentrated proteins, ash, and fibers in the final product. The increase in protein content following lipid extraction is consistent with previous findings in edible insects, where defatting enhances protein concentration by reducing the lipid fraction of the matrix [48,49].

The higher protein levels observed in DPS suggest that ethanol is highly efficient in extracting lipids while preserving the protein fraction. However, mechanical pressing (DPP), although less efficient in removing fat, still produced a significant enrichment in protein and ash content, highlighting its potential as a clean, solvent-free method. These differences may also result from structural changes in the matrix caused by compression during pressing or solvent action on cell walls, which can affect the integrity of proteins and the accessibility of nutrients [24].

Moisture content was also influenced by the defatting method. DPS samples retained more moisture, likely due to increased hydrophilicity following lipid removal. Solvent extraction removes hydrophobic lipids and disrupts lipid–protein interactions, exposing polar functional groups (e.g., hydroxyl, amino, and carboxyl), which increase the matrix’s affinity for water [26,50]. In contrast, DPP samples were drier, possibly due to compression forces reducing water retention. This suggests that solvent extraction not only alters composition but also affects the matrix’s hydration capacity.

The progressive increase in ash content with defatting supports the hypothesis that mineral components become more concentrated as lipids are removed. Similarly, the fiber content increase in DPS can be attributed to the removal of the lipid fraction, which elevates the relative concentration of non-lipid constituents, including fibers. Regarding caloric value, the significant reduction observed in DPS makes it a favorable ingredient for protein-enriched, low-calorie formulations. Conversely, the higher caloric density of FP and DPP samples may be advantageous in applications where energy content is desirable.

Water activity (Aw) results reflect differences in matrix interactions with moisture. The drastic reduction in Aw in DPS may be due to the removal of lipids and polar compounds such as phospholipids and hydrophilic proteins that typically interact with water. Lower Aw enhances product stability by reducing microbial and oxidative risks, an essential factor for extending shelf life [51].

Color parameters revealed that solvent extraction lightened the powder, reducing redness (a*) and increasing yellowness (b*), possibly due to the removal or alteration of pigments and polyphenol–protein complexes. These findings align with previous reports by Borremans et al. [52], which indicate that defatting can reduce the dark coloration of insect powders, making them more visually appealing in food formulations [53].

**Figure 2:**
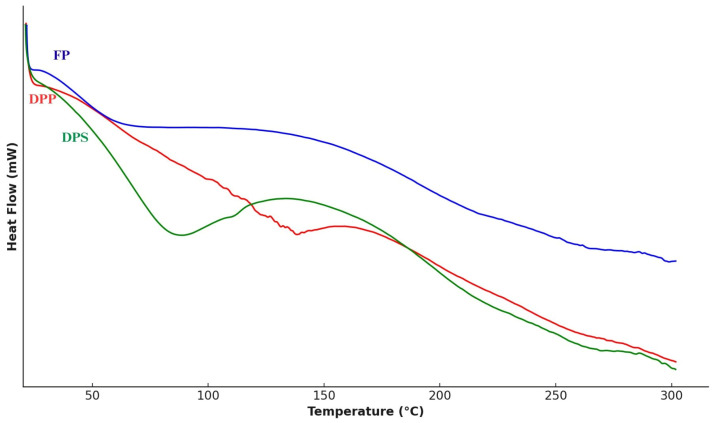
Differential scanning calorimetry (DSC) thermograms of *Hermetia illucens* larvae powders. FP = full powder form; DPP = defatted powder by pressing; DPS = defatted powder by solvent.

Thermal properties assessed by DSC indicated structural differences among samples. The higher enthalpy in DPS may be related to stronger water–protein interactions and the presence of polar groups exposed after defatting (Figure 2). In contrast, DPP samples, subjected to mechanical pressure, showed lower evaporation temperatures, possibly due to microstructural changes that facilitated water diffusion. The intact sample (FP) retained higher evaporation temperatures, likely due to the lipid barrier limiting water mobility. Overall, the defatting method plays a critical role not only in nutritional composition but also in techno-functional and thermal behavior of BSF powder, influencing its potential applications in food systems [54,55].

### 4.2. Functional and Technological Properties

Water-holding capacity (WHC) is a key functional property in food systems, as it affects moisture retention, texture, and palatability. The higher WHC observed in the DPS sample may be attributed to protein denaturation and the exposure of hydrophilic groups following solvent extraction. This structural unfolding facilitates stronger interactions with water. In contrast, the lower WHC in the DPP sample suggests a more compact matrix with fewer accessible polar groups, possibly due to the mechanical compression during pressing [56].

Oil-holding capacity (OHC) did not differ significantly between the samples, indicating that defatting, regardless of method, did not drastically alter the number of non-polar protein sites responsible for oil absorption. As OHC is largely governed by capillary action and hydrophobic interactions, the preservation of these regions in all treatments suggests that BSF powders could be suitable for formulations with moderate lipid requirements, such as baked goods and sausages [57].

The emulsification capacity (EC) was notably higher in the defatted samples, particularly DPS. This enhancement may be due to improved surface activity and increased exposure of hydrophobic and charged regions on the protein surface after lipid removal. These properties facilitate better interaction at the oil-water interface, promoting emulsification. Moreover, solvent extraction may lead to more pronounced structural unfolding than pressing, enhancing protein dispersion in aqueous media [58,59].

Emulsion stability (ES) was similar for DPS and FP, both outperforming DPP. This suggests that the interaction between oil and protein was more favorable in the solvent-defatted powder and in the intact sample, potentially due to electrostatic repulsion among oil droplets and partial protein denaturation that exposes amphiphilic amino acids [52]. These results highlight DPS as a promising ingredient in emulsified food systems, potentially serving as a substitute for conventional emulsifiers like casein or whey [60].

DPS also showed superior water solubility index (WSI) and swelling capacity (SC), which are desirable properties in products such as instant soups, beverages, or rehydratable meals. The solvent-based defatting process likely disrupted lipid–protein interactions, exposing more polar sites and enhancing water absorption. This structural modification facilitates hydration and swelling, improving functionality in aqueous environments [50].

Interestingly, DPP exhibited the highest hygroscopicity after 24 h and 7 days. This may result from a combination of preserved protein integrity and increased surface accessibility, allowing moisture uptake from the environment. Hygroscopicity is a double-edged property: it can enhance enzymatic digestibility and rehydration but may also reduce shelf life due to moisture sensitivity. In contrast, the lower hygroscopicity in FP and DPS suggests improved storage stability but potentially reduced bioavailability or digestibility in certain applications [3].

Altogether, these findings reinforce that defatting not only alters the composition of BSF powder but also modifies its functional properties. While solvent extraction enhances solubility, emulsification, and water retention, mechanical pressing preserves hygroscopicity and some structure-related characteristics. Thus, the choice of defatting method should align with the intended functional role of the ingredient in food formulations.

### 4.3. Bioactive Compounds

The antioxidant activity observed in both defatted samples (DPP and DPS) demonstrates that lipid removal may favor the release or preservation of antioxidant constituents in BSF powder. Among the treatments, the pressing method (DPP) led to the highest antioxidant capacity, suggesting that mechanical extraction better preserves thermolabile or solvent-sensitive compounds. These findings are supported by studies indicating that pressing, despite yielding lower oil recovery than solvents, tends to retain more antioxidant peptides and phenolic-like compounds in protein-rich by-products [61,62].

In contrast, although DPS involved more complete lipid extraction, its antioxidant activity was not superior to DPP. This may be due to degradation of bioactive compounds by ethanol or due to the removal of lipid-soluble antioxidants. Moreover, mechanical pressure might facilitate the release of matrix-bound antioxidants by disrupting structural barriers, which could partially explain the enhanced activity in DPP. These results are particularly relevant for the development of functional foods and supplements, as antioxidant-rich powders may contribute to oxidative stability and health-promoting properties, including the mitigation of oxidative stress and inflammation [63].

Phytochemical screening revealed that defatting increased the detectability of several classes of secondary metabolites, notably flavonoids, flavanones, and coumarins. These compounds were detected predominantly in DPP and DPS, and not in the intact powder (FP), suggesting that lipid removal facilitates solvent access and extraction efficiency. This finding is consistent with the hypothesis that the lipid matrix may act as a physical barrier limiting solute diffusion and solvent penetration. Interestingly, ethanol extraction yielded a higher concentration of phytochemicals compared to methanol in most cases. However, aqueous extracts performed similarly or better for some classes of compounds. This may be attributed to the animal origin of the raw material, which differs in matrix composition from plant tissues, where organic solvents are traditionally more effective [64].

The absence of tannins, quinones, and phenols, and the exclusive detection of steroids in aqueous extracts, suggests a selective distribution of these compounds in BSF and possibly a lower concentration in the cuticle or internal tissues compared to plants. The presence of these compounds only in defatted samples also underscores the importance of processing conditions for phytochemical accessibility. These findings contribute to the growing interest in edible insects as sources of bioactive substances, even though current literature on insect phytochemicals remains scarce. The detection of flavonoids and terpenes in BSF aligns with the hypothesis that insects may bioaccumulate phytochemicals from their diet or synthesize analogs with similar biological functions. Such compounds are known for their antioxidant, anti-inflammatory, and prebiotic properties, and regular intake has been associated with improved immune function, reduced oxidative stress, and enhanced metabolic health [65,66,67,68,69].

Overall, the results reinforce the potential of defatted BSF powders, especially those obtained through mechanical pressing, as functional ingredients rich in bioactive compounds. These powders may be suitable for applications in foods, nutraceuticals, or cosmetic products where antioxidant protection and health benefits are desirable.

## 5. Conclusions

This study highlights the significant influence of defatting methods on the nutritional, functional, and bioactive properties of *Hermetia illucens* larvae powder. Both mechanical pressing and ethanol-based solvent extraction yielded powders with potential for various applications, including high-protein ingredients, bioactive compound extraction, and sustainable food formulations. Solvent extraction was more effective in lipid removal, reducing fat content from 32.45 to 3.18 g/100 g and increasing protein concentration from 34.13 to 54.96 g/100 g. It also improved water solubility and swelling capacity, and reduced water activity to 0.269—properties desirable for protein enrichment and emulsified food systems. However, this method may lead to the loss of certain antioxidant compounds, such as saponins, and requires careful handling due to the use of chemical solvents.

In contrast, mechanical pressing, while less efficient in removing lipids (final fat content: 21.70 g/100 g), better preserved bioactive compounds and resulted in the highest antioxidant activity (68.30% DPPH inhibition). This suggests its suitability for health-oriented products where oxidative stability and bioactivity are desirable. Ultimately, the choice of defatting method should be guided by the intended application, balancing nutritional enhancement and functional performance.

These findings provide a framework for the tailored development of insect-based powders and support the broader integration of edible insects into sustainable and circular food systems. Specifically, they reinforce the potential of *Hermetia illucens* as a scalable and innovative solution for sustainable protein production.

## Figures and Tables

**Table 1:** Result of physicochemical and thermal characterization of BSF samples.

Parameter		FP	DPP	DPS
Moisture (g/100 g)		6.07 ± 0.10 ^b^	5.20 ± 0.17 ^c^	7.01 ± 0.32 ^a^
Protein * (g/100 g)		34.13 ± 2.05 ^c^	47.16 ± 2.73 ^b^	54.96 ± 1.26 ^a^
Lipids *(g/100 g)		32.45 ± 4.53 ^a^	21.70 ± 1.39 ^b^	3.18 ± 0.55 ^c^
Crude fiber * (g/100 g)		8.87 ± 0.62 ^b^	7.73 ± 0.35 ^c^	13.90 ± 0.43 ^a^
Ash * (g/100 g)		8.58 ± 0.08 ^c^	9.42 ± 0.15 ^b^	10.70 ± 0.04 ^a^
Carbohydrates * (g/100 g)		10.61	11.37	10.45
Energy value (kcal/100 g)		471.01	407.37	288.64
Aw		0.571 ± 0.009 ^a^	0.529 ± 0.006 ^a^	0.269 ± 0.060 ^b^
L*		31.88 ± 0.44 ^b^	33.03 ± 0.73 ^b^	57.09 ± 0.13 ^a^
a*		9.54 ± 0.17 ^a^	9.79 ± 0.20 ^a^	5.92 ± 0.35 ^b^
b*		22.53 ± 0.44 ^b^	23.40 ± 0.26 ^b^	24.01 ± 0.93 ^a^
h (°)		67.22 ± 0.10 ^b^	67.04 ± 0.32 ^b^	76.15 ± 0.29 ^a^
Thermal properties	T_p_ (°C)	138.30	54.15	88.88
	T_0_ (°C)	128.34	49.46	62.11
	T_f_ (°C)	144.28	80.19	118.26
	ΔH (J/g)	8.77	8.37	108.66

FP = full powder form; DPP = defatted powder by pressing; DPS = defatted powder by solvent; Aw = activity water. * Dry basis. T_0_ = onset temperature; T_p_ = peak temperature; T_f_ = final temperature; ΔH = enthalpy. The average of three values with standard deviation; the same letter in the line indicates that there were no significant differences between the means, according to Tukey’s test (*p* < 0.05).

**Table 2:** Antioxidant activity of BSF larvae samples in full powder form (FP), defatted powder by pressing (DPP), and defatted powder by solvent (DPS).

	Antioxidant Activity (% Inhibition)	
	ABTS Radical Capture	DPPH Radical Sequestration
FP	45.43 ± 0.44 ^b^	52.54 ± 0.27 ^b^
DPP	48.00 ± 1.20 ^a^	68.30 ± 0.25 ^a^
DPS	46.93 ± 1.31 ^ab^	54.12 ± 1.53 ^ab^

FP = full powder form; DPP = defatted powder by pressing; DPS = defatted powder by solvent. Average of three values with standard deviation; the same letter in the column indicates that there were no significant differences between the means according to Tukey’s test (*p* < 0.05).

**Table 3:** Phytochemical profile of BSF samples.

Components	FP			DPP			DPS		
	Water	Methanol	Ethanol	Water	Methanol	Ethanol	Water	Methanol	Ethanol
Coumarins	(+)	(−)	(−)	(+)	(+)	(+)	(+)	(+)	(+)
Flavonoids	(+)	(−)	(−)	(+)	(+)	(+)	(+)	(+)	(+)
Saponins	(−)	(+)	(+)	(−)	(−)	(+)	(+)	(−)	(+)
Flavanones	(−)	(−)	(−)	(+)	(+)	(+)	(+)	(+)	(+)
Steroids	(+)	(−)	(−)	(+)	(−)	(−)	(+)	(−)	(−)
Tannins	(−)	(−)	(−)	(−)	(−)	(−)	(−)	(−)	(−)
Quinone	(−)	(−)	(−)	(−)	(−)	(−)	(−)	(−)	(−)
Phenols	(−)	(−)	(−)	(−)	(−)	(−)	(−)	(−)	(−)

Symbol (−) indicates the absence of specific phytochemicals, whereas (+) indicates their presence. FP = full powder form; DPP = defatted powder by pressing; DPS = defatted powder by solvent.

## Data Availability

The original contributions presented in this study are included in the article. Further inquiries can be directed to the corresponding author.

## Abbreviations

The following abbreviations are used in this manuscript:

FP	Full powder form
DPP	Defatted powder by pressing
DPS	Defatted powder by solvent
